# CIN2 + detection in high-risk HPV patients with no or minor cervical cytologic abnormalities: a clinical approach validated by machine learning

**DOI:** 10.1007/s00404-023-06953-6

**Published:** 2023-02-13

**Authors:** Julia Wittenborn, Tomas Kupec, Séverine Iborra, Laila Najjari, Lieven N. Kennes, Elmar Stickeler

**Affiliations:** 1 Department of Obstetrics and Gynecology of the University Hospital Aachen, Pauwelsstr. 30, 52074 Aachen, Germany; 2 Department of Economics and Business Administration, University of Applied Sciences Stralsund, Zur Schwedenschanze 15, 18435 Stralsund, Germany

**Keywords:** Machine learning, ASC-US, NILM, Colposcopy

## Abstract

**Purpose:**

To evaluate the feasibility and diagnostic value of the combination of colposcopy, cytology and hrHPV (high-risk human papilloma virus) PCR (polymerase chain reaction) testing in patients with no or minor cytologic abnormalities and HPV high risk infection and to find the best predictors for the presence of CIN2 + in this patient collective.

**Methods:**

Three hundred and thirty-four hrHPV patients with normal cytology or minor cytologic abnormalities who had a colposcopic examination at the center of colposcopy at the university hospital Aachen in 2021 were enrolled in this retrospective cohort analysis. Multivariate logistic regression and a machine-learning technique (random forests, leave-one-out analysis) were used.

**Results:**

The overall risk for CIN2 + in hrHPV-positive patients with normal cytology was 7.7% (*N* = 18) (5% for CIN3 +), 18% (*N* = 16) (10.1% for CIN3 +) in patients with PAP IIp (ASC-US) and 62.5% (*N* = 5) (25% for CIN3 +) in patients with PAP IIg (AGC). Variables that show a statistically significant influence for the CIN-status are ‘major change’ as the result of colposcopy, transformation zone type T1, PAP IIg upon referral (AGC) and hrHPV category 1a (HPV 16/18) detection. Using machine learning (random forests) techniques, the main influencing variables were confirmed. A monotonously decreasing risk for CIN2 + from hrHPV category 1a to 3 (in accordance to the IACR guidelines) was found.

**Conclusion:**

In the collective of hrHPV patients with no or minor cytologic abnormalities, the result of colposcopy and HPV PCR status are key predictors for the detection of CIN2 + with a monotonously decreasing risk for CIN2 + from hrHPV category 1a to 3.

## What does this study add to the clinical work

**Table:** 

In the collective of hrHPV (high risk human papilloma virus) patients with no or minor cytologic abnormalities, besides the colposcopic result, HPV PCR status is a key predictor for CIN2+ detection with a monotonously decreasing risk for CIN2+ from hrHPV category 1a to 3. For a differential approach in cervical cancer screening depending on the detected HPV subtype, it is crucial to perform HPV tests with detection of different high risk genotypes in clinical practice. Patients with hrHPV category 3 should not be referred for colposcopy.	

## Introduction

Cervical cancer is the fourth most common tumour in women worldwide [1]. Human high-risk papillomavirus (hrHPV), especially in persistent infections, causes cervical dysplasia (also known as cervical intraepithelial neoplasia [CIN]) and consecutively cancer [2–4]. Although the majority of women with a hrHPV infection will never develop CIN or cancer, a relatively large number of women is at risk of developing CIN. Nearly, all developed countries implemented cervical cancer screening programs in order to reduce the incidence of cervical cancer [5–13]. Due to the introduction of a program in the 1970s cervical cancer is a rare disease in Germany today and only about 4300 patients are diagnosed with cervical cancer every year [14]. Due to overwhelming evidence from long-term prospective cohorts and randomized clinical trials demonstrating that hrHPV DNA testing is considerably more sensitive than cervical cytology for the detection of cervical intraepithelial neoplasia grade and cancer, a new cervical cancer screening algorithm was implemented in 2020 [15–21]. HrHPV testing was introduced as a supplementary test in patients with 35 years and older. As hrHPV testing is widely used, the optimal clinical management of women with hrHPV infection, especially combined with no or minor cytological abnormalities, remains a challenge. According to the German national guidelines, patients with one-time low-grade cytologic abnormalities (ASC-US) are referred to colposcopy if they carry a hrHPV infection. Also, patients with persistent hrHPV infection over one year and normal cytology are referred [22]. As hrHPV has a high population prevalence [23], this new patient collective in the German cervical cancer screening has led to a high influx of patients in all centers for colposcopy. The challenge in the future development of cervical cancer screening programmes is to reach an optimum balance between benefit, harm and affordability.

Here, we present a study showing the incidence rates of CIN2 + and CIN3 + in the new cervical cancer screening cohort of patients with normal or no cytologic abnormalities and HPV high-risk infection. We evaluated the feasibility and diagnostic value of the combination of colposcopy, cytology and hrHPV PCR (polymerase chain reaction) testing and aimed to find the best predictors for the presence of CIN2 + using logistic regression and machine learning methods.

## Methods

### Study population

The study was designed as a retrospective cohort study of all patients referred to the center of colposcopy at the university hospital in Aachen in 2021 with normal (NILM) or minor (ASC-US, AGC) cervical cytological abnormalities and HPV high risk infection upon referral. In 2021 started the high influx of patients with normal cytologies to centers for colposcopy. Even though the new screening algorithm was implemented in 2020, women with normal cytology were only referred for colposcopy if they showed a persistently positive HPV test after one year.

### Data collection

In our department, colposcopies are performed in standardized conditions using a Leisegang 3MCV colposcope. The general assessment was carried out in accordance with the 2011 International Federation for Cervical Pathology and Colposcopy (IFCPC) colposcopic terminology for the cervix: transformation zone types were classified accordingly as 1, 2 or 3. Although all patients included in the study had a PAP smear and HPV testing upon referral, a conventional control Pap smear of the cervix, a control test for human papillomavirus (PCR for HPV DNA) and application of 5% acetic acid to the cervix represent the standard of care in our unit, and this procedure was carried out for every woman who was referred for colposcopy. The use of Lugol’s iodine solution was not routinely applied and its use remained at the examiners discretion. During the whole period of investigation, the Seegene Anyplex II HPV 28 detection kit was used. It simultaneously detects 19 high-risk and intermediate-risk HPV genotypes and 9 low-risk types and is certified for the use in cervical cancer screening [24, 25]. The classification of the HPV virus to the different categories was in accordance with the IACR (international Association of cancer registries) guidelines. Category 1a included HPV 16 and 18. HPV 31/33/35/52/58/45 were comprised in category 1b. Category 2 included HPV 51/56/39/59 and category 3 HPV 68/73/66. The detection of multiple HPV high-risk viruses was defined as HPV high risk multiple infection. The colposcopic findings were classified in accordance with the IFCPC into “normal,” “minor,” “major,” and “suspicious for invasion/cancer.” Normal findings included, for example, metaplasia, viral warts, or polyps. Minor findings are defined by delicate puncturing, thin acetowhite epithelium, and irregular and geographic borders. Typical major lesions are represented by sharp borders, an inner border, ridge sign, dense acetowhite epithelium, a coarse mosaic pattern, and coarse puncturing. Atypical vessels, fragile vessels, irregular surface, exophytic lesions, necrosis, and ulceration are suspicious for invasion [26]. A colposcopy directed biopsy was taken in all cases. In patients with T1 transformation zone, it was taken from the most suspicious part of the acetowhite area. In case of T3 transformation zone, an endocervical curettage was performed. In some patients with multifocal lesions, more than one biopsy was taken. At least a slight acetowhite reaction was observed in all patients; thus, all patients included in the study had a histological work-up. The known risk factors for the presence of cervical intraepithelial dysplasia were collected for every patient (see Table 1), e.g., smoking, history of LEEP and patients’ age. All colposcopies were performed by experienced, highly qualified and AG CPC certified staff at the DKG certified colposcopy unit at the University Hospital Aachen. Decisions regarding surgical treatment were based on the German S3 guideline for prevention of cervical cancer [27].

**Table 1:** Patient characteristics

	Total number (*N* = 334)
Cytology upon referral	
PAP I	233 (69.8%)
PAP IIa	4 (1.2%)
PAP IIg	8 (2.4%)
PAP IIp	89 (26.7%)
Control cytology	
PAP I/IIa(NILM)	189 (57%)
PAP IIg (AGC)	3 (0.9%)
PAP IIp(ASC-US)	112 (33.5%)
PAP IIID1 (LSIL)	14 (4.2%)
PAP IIID2 (HSIL)	10 (3%)
PAP IIIp (ASC-H)	3(0.9%)
PAP IVap (HSIL)	3 (0.9%)
Result of control HPV PCR	
Neg 0	70 (20.1%)
HPV 16 or 18 (category 1a)	77 (23%)
HPV 31/33/35/52/58/45 (categorie 1b)	102 (31%)
HPV 51/56/39/59 (category 2)	57 (17.1%)
HPV 68/73/66 (category 3)	26 (7.8%)
HPV High-risk multiple infection	
Yes	75 (22.5%)
No	256 (76.7%)
Missing	3
Age	
Median	47
Mean ± SD	48.57 ± 10.78
Min	26
Max	83
Smoking	
Yes	83 (24.9%)
No	84 (25.2%)
History of smoking	51(15.3%)
Unknown	116 (34.7%)
History of HPV	
History of HPV category 1	39 (11.7%)
History HPV category 2/3	9 (2.7%)
History of HPV high risk (unspecified)	35 (10.5%)
No history of HPV	251 (75.2%)
History of cytological abnormalities	
Yes	19 (5.7%)
No	315 (94.3%)
HPV Low Risk Infection	
Yes	49 (14.7%)
No	282 (84.4%)
Colposcopy	
Minor change	159 (47.6%)
Major change	27 (8.1%)
Normal	146 (43.7%)
Inadequate	2 (0.6%)
Localization of transformation zone	
T1	63 (18.9%)
T2	62 (18.6%)
T3	209 (62.6%)
Result of colposcopy-directed biopsies	
Normal /no evidence of dysplasia	271 (81.1%)
CIN 1	24 (7.2%)
CIN 2	16 (4.8%)
CIN 3	23 (6.9%)
Localization of CIN 2 +	
Ektocervical	8 (20.5%)
Endocervical	31 (79.5%)
Intrauterine device	
Yes	10 (3%)
No	324 (97%)
Pervious conization	
Yes	24 (7.2%)
No	310 (92.8%)
Presence of a cervical polyp	
Yes	11 (3.3%)
No	323 (96.7%)

### Ethical approval

The study was approved by the Ethics Committee at the RWTH Aachen University Faculty of Medicine, Germany in January 2022 (EK 011/22). All procedures performed in this study involving human participants were in accordance with the ethical standards of the institutional and/or national research committee and with the 1964 Helsinki Declaration and its later amendments.

### Statistical analysis

Continuous variables are expressed as mean values ± standard deviation (SD). Categorical data are presented by absolute frequencies and percentages. Differences in each variable between CIN-status (CIN2 + or Non-CIN2 +) groups were summarized by descriptive statistics. The association between two categorical variables was investigated by contingency tables.

### Inferential statistics and predictive modelling

To identify the most meaningful predictor variables for CIN2 + and to classify patients according to these predictor variables, multivariate logistic regression as well as random forest was conducted.

### Multivariate logistic regression

Multivariate logistic regression including all potential variables was performed to investigate their influence on the CIN status. The model selection was performed using the Akaike information criterion (AIC) [28]. The AIC is defined by $2k-2ln(L)$, where $k$ is the number of predictor variables and $L$ is the maximum value of the likelihood function of the logistic regression model. The better the model fits the data, the higher is the value of the likelihood function and thus the lower is the AIC. Models with lower AIC are better. The number of predictor variables is entering the AIC positively, thus the $2k$-term is often referred to as a “penalty term”, penalizing the addition of extra variables discouraging overfitting. The best-fitting model, with the lowest AIC, is reported in the results section.

To assess performance of prediction for unseen data, the ‘Leave-one-out’-method was used: For each subject, the logistic regression model was derived based on the remaining 333 subjects and the resulting model utilized to predict the probability of CIN2 + for the subject, who was left out during the training. If this probability was above 30% (due to imbalance data), the subject was classified as CIN2 + , otherwise as Non-CIN2 + . This procedure was repeated for all 334 subjects. The relative frequency of correct classifications is referred to as the accuracy. To make results comparable to the machine learning (ML) procedure random forest, this standard ML-performance metrics was derived. To correct for imbalanced data (Non-CIN2 + : 295, CIN2 + : 39), additionally to accuracy, the balanced accuracy was derived. The balanced accuracy is the mean of the two relative frequencies of positive and negative cases identified correctly.

### Machine learning

As second method, machine learning, more specifically random forest, was used for the identification of meaningful predictor variables and risk estimation of CIN2 +. The advantage of random forest compared to multivariate logistic regression is that random forest also recognizes non-linear relationships.

A random forest with 500 trees was applied on the data. The 500 random samples of the usual 63.2% of observations were used to fit 500 decision trees. Additionally, variable importance was assessed to determine how important different variables are for classifying the CIN status correctly. As the regular CART trees are biased towards continuous variables and variables with many categories, unbiased conditional inference trees were used to account for the situation of different variable types [29]. Due to imbalanced data, weights according to the relative frequencies of CIN status were used in the random forest. The same performance metrics as for logistic regression were assessed for random forest (i.e., accuracy and balanced accuracy). Again, to assess performance of prediction for unseen data, the ‘Leave-one-out’-method was used.

### General

*P* < 0.05 was considered statistically significant. Because of the exploratory nature of the study the significance level was not adjusted for multiplicity. The statistical analyses were conducted using the statistical software R [30].

## Results

One thousand four hundred eighty-one patients who presented to our center for colposcopy in the year 2021 were assessed for eligibility. A total of 334 patients with normal cytology (NILM, PAP I/IIa) or minor cytologic abnormalities (ASC-US, PAP IIp or AGC, PAP IIg) upon referral were included in the study. 237 had hrHPV-positive/cytology-negative results after return testing at 1 year and 97 had low grade cytologic abnormalities (ASC-US, AGC) and hr HPV positivity and were therefore referred for a colposcopic examination in 2021. The result of the colposcopy-directed biopsy or the endocervical curettage (in case of T3 transformation zone) was normal in 81.1% (*N* = 271). CIN 1 was detected in 7.2% (*n* = 24), CIN2 in 4.8% (*n* = 16)and CIN3 in 6.9% (*n* = 23). The localization of CIN2 + was ectocervical in 79.5% and endocervical in 20.5%. The patient’s characteristics are displayed in Table 1 as well as the results of the control cytology and the result of the control HPV PCR. 20% of all patients had a negative control HPV PCR. Table 2 shows the comparison of cytology upon referral and control cytology. Median Age was 48.57 ± 10.78 years. The majority of included patients had a T3 transformation zone (62.6%). The result of the colposcopic examination was normal in 43.7% of cases and revealed minor or major changes in 47.6% and 8.1% of cases, respectively. 77% of the patients with CIN2 + received a loop electrical excision procedure of the cervix (LEEP). In 56.7% of the cases CIN2 + was also found in the histopathological workup of the LEEP, and in 43.3% no residual disease was detected.

**Table 2:** Comparison of cytology upon referral and control cytology

	PAP I/IIa	IIg	IIID1 (LSIL)	IIID2 (HSIL)	IIIp (ASC-H)	IIp (ASC-US)	IVap (HSIL)
PAP I (NILM)	152	1	8	6	3	62	1
PAP IIa (NILM)	3	0	0	0	0	1	0
PAP IIg (AGC)	3	1	1	2	0	0	1
PAP IIp (ASCUS)	31	1	5	2	0	49	1

The results of the bivariate analysis are displayed in Table 3. In 7.6% (*N* = 18) of the hr HPV-positive patients with normal cytology upon referral, a CIN2 + was detected in the colposcopy-directed biopsies. In the group of patients with low-grade cytologic abnormalities, 18% (*N* = 16) of patients with PAP IIp (ASC-US) had a CIN2 + and 62.5% (*N* = 5) of patients with PAP IIg (AGC). Looking at hrHPV infection, in category 1, 19.5% (*N* = 15) of the patients had a CIN2 +, in category 1b 14.7% (*N* = 15), in category 2 10.5% (*N* = 6) and none in category 3. 16% of the patients with HPV high-risk multiple infection had a CIN2 + and 10.5% with no HPV high-risk multiple infection.

**Table 3:** Results of the bivariate statistical analysis using CIN2 + as positive endpoint

	Neg (Non-CIN2 +)	Pos (CIN2 +)
Cytology upon referral		
PAP I/IIa	219 (92.4%)	18 (7.6%)
PAP IIg	3 (37.5%)	5 (62.5%)
PAP IIp	73 (82.0%)	16 (18.0%)
Control cytology		
PAP I/IIa	184 (97.4%)	5 (2.7%)
PAP IIp	96 (85.7%)	16 (14.3%)
PAP IIg / IIID1 (LSIL)	9 (52.9%)	8 (47.1%)
PAP IIID2 /IIIp /IV ap	6 (37.5%)	10 (62.5%)
Result of HPV PCR		
Neg 0	67 (95.7%)	3 (4.3%)
HPV 16 or 18 (category 1a)	62 (80.5%)	15 (19.5%)
HPV 31/33/35/52/58/45 (categorie 1b)	87 (85.3%)	15 (14.7%)
HPV 51/56/39/59 (category 2)	51 (89.5%)	6 (10.5%)
HPV 68/73/66 (category 3)	26 (100%)	0
Missing	2	0
HPV High-risk multiple infection		
Yes	63 (84.0%)	12 (16.0%)
No	229 (89.5%)	27 (10.5%)
Age		
Mean	49.1	44.5
Smoking		
Yes	69 (83.1%)	14 (16.9%)
No	77 (91.7%)	7 (8.3%)
History of smoking	45 (88.2%)	6 11.9%)
Unknown	104 (89.7%)	12 (10.3%)
History of HPV		
History of HPV category 1	34 (87.2%)	5 (12.8%)
History HPV category 2/3	9 (100%)	0
History of HPV high risk (unspecified)	33 (94.3%)	2 (5.7%)
No History of HPV	219 (87.3%)	32 (12.8%)
History of cytological abnormalities		
No	281 (89.2%)	34 (10.8%)
Yes	14 (73.7%)	5 (26.3%)
HPV low risk infection		
Yes	45 (91.8%)	4 (8.2%)
No	247 (87.6%)	35 (12.4%)
Colposcopy		
Minor change	142 (89.3%)	17 (10.7%)
Major change	9 (33.3%)	18 (66.7%)
Normal	142 (97.3%)	4 (2.7%)
Inadequate	2	0
Localization of transformation zone		
T1	44 (69.9%)	19 (30.1%)
T2	52 (83.9%)	10 (16.1%)
T3	199 (95.2%)	10 (4.8%)
Intrauterine device		
Yes	9 (90.0%)	1 (10.0%)
No	286 (88.3%)	38 (11.7%)
Pervious conization		
Yes	21 (87.5%)	3 (12.5%)
No	274 (88.4%)	36 (11.6%)
Presence of a cervical polyp		
Yes	8 (72.7%)	3 (27.3%)
No	287 (88.9%)	36 (11.1%)

Regarding the results of the colposcopic examination, in patients with major change lesion a CIN2 + was detected with a relative frequency of 66.7%. If colposcopy revealed minor change lesions, 10.7% were detected and in 2.7% of patients with a normal colposcopy. As can be seen in Table 4, most cases resulting in normal or minor change colposcopy were patients with T3 transformation zone.

**Table 4:** Comparison of the result of the colposcopic examination and localization of the transformation zone

	Major change	Minor change	Normal
T11	**14 (22%)**	41 (65%)	8 (13%)
T2	8 (13%)	41(66%)	13 (21%)
T3	5 (2%)	77 (37%)	**125 (60%)**

In patients with T1 transformation zone, a CIN2 + was detected in 30.1% (*N* = 19), in patients with T2 transformation zone in 16.1% (*N* = 10) and in patients with T3 transformation zone in 4.8% (*N* = 10).

Table 5 shows the results of the bivariate analysis using CIN3 + as outcome parameter. 5% of patients with normal cytology upon referral had CIN3 + (*N* = 12), 10.1% (*N* = 9) of patients with PAP IIp (ASC-US) and 25% (*N* = 2) of patients with PAP IIg (AGC).

**Table 5:** Results of the bivariate statistical analysis using CIN3 + as endpoint

	Neg (non CIN3 +)	CIN3 +
Cytology upon referral		
PAP I /IIa	225 (95.0%)	12 (5.0%)
PAP IIg	6 (75.0%)	2 (25.0%)
PAP IIp	80 (89.9%)	9 (10.1%)
Control cytology		
PAP I/IIa	186 (98.4%)	3 (1.6%)
PAP IIg / IIID1 (LSIL)	13 (76.5%)	4 (23.5%)
PAP IIp	102 (91.1%)	10 (8.9%)
PAP IIID2 /IIIp /IV ap	10 (62.5%)	6 (37.5%)
Result of HPV PCR		
Neg (category 0)	68 (97.1%)	2 (2.9%)
HPV 16 or 18 (category 1a)	64 (83.1%)	13 (16.9%)
HPV 31/33/35/52/58/45 (categorie 1b)	96 (94.1%)	6 (5.9%)
HPV 51/56/39/59 (category 2)	55 (96.5%)	2 (3.5%)
HPV 68/73/66 (category 3)	26 (100%)	0
Missing		

The results of the multivariate analysis using logistic regression are displayed in Table 6. As control cytology and colposcopy-directed biopsies were often analysed by the same pathologist, control cytology was removed from the analysis because of potential bias and strong collinearity. Model selection based on the AIC [28] revealed the variables contributing sufficiently to the model (displayed in Table 6). Variables that show a statistically significant influence for the CIN status as the result of the colposcopy-directed biopsies are highlighted. Based on the final multivariate logistic regression model, ‘major change’ as the result of colposcopy had the main influence on the CIN status (*p* < 0.0001). Other variables with statistically significant influence on the CIN status were: the change from transformation zone type T3 to T1 and PAP IIg upon referral (AGC) and hrHPV category 1a (HPV 16/18) detection. The accuracy of this model in predicting CIN2 + is 91%. As both groups are unbalanced in the number of patients, we also calculated a balanced accuracy, which is 75%.

**Table 6:** Results of logistic regression

	Estimates	*p*-values
T1 transformation zone	**1.4387**	**0.00821**
T2 transformation zone	0.3844	0.52578
PAP IIg upon referral	**2.8218**	**0.00190**
PAP IIp upon referral	0.5701	0.23730
Colposcopic major change lesion	**3.4870**	** < 0.0001**
Colposcopic minor change lesion	0.8640	0.17449
HPV category 1a	**7.9650**	**0.01433**
HPV category 1b	1.4496	0.06620
HPV category 2	1.4584	0.09646

Significant results (*p*< 0.05) are printed bold

In the random forest analysis, the result of colposcopy (major change) and transformation zone (type 1) were the most important variables, with an accuracy of 91% and balanced accuracy of 71%.

The accuracy for unseen data is 90% for both the random forests analysis and logistic regression analysis. The balanced accuracy is 70 and 72%, respectively.

## Discussion

Since the introduction of the new cervical cancer screening in Germany, the rising numbers of colposcopies have been in the focus of discussion. Hr HPV-positive patients with normal cytology or minor cytologic abnormalities comprise a group of patients now in need of colposcopy according to the German national guidelines [22]. As hrHPV testing is widely used, the optimal clinical management of women with hrHPV infection, especially combined with no or minor cytological abnormalities, remains a challenge.

Here, we present data from our study using the combination of colposcopy, control cytology, colposcopy-directed biopsies and hrHPV testing (PCR) in detecting CIN2 + in patients with no or minor cytologic abnormalities.

Previous studies have mostly been concerned with concordance rates between the different diagnostic tools and respective consistency rates [18, 31–34]. In clinical practice, it is the combination of all diagnostic tools that is routinely used and we sought to find the best predictors of CIN2 + in the patient collective of hrHPV-positive/cytology negative or low-grade cytologic abnormalities.

The most important predicting variables were the colposcopic presence of a major change lesion, T1 transformation zone, AGC (PAP IIg) as cytology upon referral and hrHPV category 1a infection. A combined accuracy rate of over 90% was achieved. As the two classes (non-CIN2 + and CIN2 +) had quite different numbers of observations, we also calculated the balanced accuracy rate, which was still 75% in the logistic regression model. We were able to confirm the main predicting variables in the random forests analysis, which shows the robustness of our results. Using the leave-one-out-analyses, we were able to assess performance metrics for unseen data for both techniques (logistic regression and random forest). The accuracies for unseen data were still above 90% for both techniques and the balanced accuracies 72% and 70% for logistic regression and random forest, respectively.

Previous reports found low sensitivity rates for colposcopy in a screening population of patients without history of abnormal PAP smears and therefore did not recommend colposcopy in this patient collective [35]. In our study, the balanced accuracy rate of colposcopy, cytology and hrHPV testing was in the range of the colposcopic accuracy of previous reports [35–38]. Thus, the combination of colposcopy and hrHPV PCR testing in a hrHPV-positive patient collective of no- or low-grade cytologic abnormalities achieves similar diagnostic accuracy as colposcopy alone in a patient collective of patients with history of abnormal PAP smears.

This result is quite remarkable, especially given the fact that most of the patients had a T3 transformation zone [34, 39]. In our experience, upon splaying the cervical canal at least with a cotton swab, in many cases it is possible to detect suspect lesions even if they are localized in the cervical canal. As all patients received biopsies or an endocervical curettage, the risk of missed dysplasia is low. In 20% of the cases, CIN2 + was only detected by the performed endocervical curettage, which is routinely performed in patients with T3 transformation zone. This result emphasizes the importance of an endocervical curettage in this patient collective formerly described as patients with unsatisfactory colposcopy. The detection rates in the endocervical curettage in the patient collective of hrHPV-positive/cytology low risk or negative are in line with previous reports [40, 41].

The overall risk for CIN2 + in hrHPV-positive patients with normal cytology was 7.7% (5% for CIN3 +). In the group of hrHPV-positive patients with low-grade cytologic abnormalities the rate of CIN2 + was higher: 18% of patients with PAP IIp (ASC-US) had a CIN2 + (10.1% for CIN3 +) and 62.5% (*N* = 5) of patients with PAP IIg (AGC) (25% for CIN3 +). The presented data are useful for the calibration of our national screening program. The threshold for referring to colposcopy will be in the centre of discussion for a while since it is based on accepted cervical cancer risk, the population real-world screening data and screening capacity [42, 43].

In another report, the detection rate of CIN2 + in patients with normal cytology was higher, but that might be based on a selection bias since not all patients received colposcopy and colposcopy -directed biopsies in that study [31]. Another possible explanation is the high quality of screening cytology in the region with low rates of under-diagnosis.

Interestingly, in the majority of the cases the control cytology was worse than the cytology upon referral. We attributed this effect to the previously described phenomenon of informed cytology [44, 45]. The cytotechnician’s assessment is influenced by the knowledge of the histological sample, hrHPV status and sometimes even the reported result of the colposcopic examination.

The colposcopy was found to be normal in only 44% of the cases but the histological assessment was normal in almost twice as many cases. A colposcopy with minor changes is not found in the category “normal”. In case of minor changes, no high-grade lesion is suspected and accordingly the histological finding is normal in many of these cases as well. In our opinion, this explains the normal histological assessment in twice as many cases.

We were able to show a monotonously decreasing risk for CIN2 + and CIN3 + from hrHPV category 1a to 3. Interestingly, in hrHPV-positive patients of category 3 no CIN2 + was found at all. HrHPV 16/18 detection is a key predicting factor for the presence of CIN2 + . This finding is in line with other reports demonstrating a significantly higher risk for CIN2 + and CIN3 + in patients with HPV 16 or 18 positivity than in patient with other hrHPV [42, 46–48]. In order to calibrate the screening algorithm a differential approach depending on the detected HPV category might be useful. In our opinion, patients with hrHPV positivity of category 3 should not be referred for immediate colposcopy.

The rate of normal histological findings in the cone specimen was rather high in the studied patient collective: In 43.3%, no residual disease could be detected despite a histologically confirmed CIN2 + in the biopsy or endocervical curettage. As small colposcopic lesions are associated with absence of CIN in the LEEP [49],we assume that CIN2 + lesions in the patient collective with normal or minor cytologic abnormalities, are often very small and are frequently removed completely by the performed biopsy.

Our study has several strengths and limitations that need to be addressed. Using machine-learning techniques, we are able to present results of a state-of-the-art classification method for assessing the accuracy of diagnostic in hrHPV-positive patients with no- or low-grade cytological abnormalities confirming the importance of colposcopy and HPV testing. The included number of patients in the study is quite large but has a natural imbalance regarding the sample sizes of the endpoint (CIN2 + vs. non-Cin2 +). That fact was accounted for in the statistical analysis using weights and calculating balanced accuracy rates.

## Conclusion

In the collective of hrHPV patients with no or minor cytologic abnormalities, colposcopy can be confirmed as the most important diagnostic tool. Besides the result of the colposcopy, HPV status is a key predictor for the detection of CIN2 + with a monotonously decreasing risk for CIN2 + and CIN3 + from hrHPV category 1a to 3. For a differential approach depending on the detected HPV subtype, it is crucial to perform HPV tests with detection of different high-risk genotypes in clinical practice. As in the collective of hrHPV-positive patients of category 3 no CIN2 + lesion was found at all, these patients should not be referred for colposcopy.

## Data Availability

The datasets generated during the current study are available from the corresponding author on reasonable request.

## References

[1] Siegel RL, Miller KD, Fuchs HE (2022). Cancer statistics, 2022. CA Cancer J Clin.

[2] Ostor AG (1993). Natural history of cervical intraepithelial neoplasia: a critical review. Int J Gynecol Pathol.

[3] zur HH (2002). Papillomaviruses and cancer: from basic studies to clinical application. Nat Rev Cancer.

[4] Walboomers JM, Jacobs MV, Manos MM (1999). Human papillomavirus is a necessary cause of invasive cervical cancer worldwide. J Pathol.

[5] Yeong ML, Pringle E, Stewart J (2013). A comparison of thinprep imager-assisted with manual screening, and its place in the New Zealand cervical cancer screening program. Pathology.

[6] Palencia L, Espelt A, Rodriguez-Sanz M (2010). Socio-economic inequalities in breast and cervical cancer screening practices in Europe: influence of the type of screening program. Int J Epidemiol.

[7] Spaczynski M, Nowak-Markwitz E, Januszek-Michalecka L (2009). Women’s social conditions and their participation in cervical cancer population screening program in Poland. Ginekol Pol.

[8] Goldhaber-Fiebert JD, Denny LA, De Souza M (2009). Program spending to increase adherence: South African cervical cancer screening. PLoS ONE.

[9] Jun JK, Choi KS, Jung KW (2009). Effectiveness of an organized cervical cancer screening program in Korea: results from a cohort study. Int J Cancer.

[10] Takac I, Ursic-Vrscaj M, Repse-Fokter A (2008). Clinicopathological characteristics of cervical cancer between 2003 and 2005, after the introduction of a national cancer screening program in Slovenia. Eur J Obstet Gynecol Reprod Biol.

[11] Rebolj M, van Ballegooijen M, Berkers LM (2007). Monitoring a national cancer prevention program: successful changes in cervical cancer screening in the Netherlands. Int J Cancer.

[12] Philips Z, Avis M, Whynes DK (2006). Introducing HPV triage into the english cervical cancer screening program: consequences for participation. Women Health.

[13] Nygard JF, Nygard M, Skare GB (2006). Pap smear screening in women under 30 in the Norwegian coordinated cervical cancer screening program, with a comparison of immediate biopsy vs Pap smear triage of moderate dysplasia. Acta Cytol.

[14] RKI ZfK. Gebärmutterhalskrebs (Zervixkarzinom). Stand 06/2020. https://www.krebsdaten.de/Krebs/DE/Content/Krebsarten/Gebaermutterhalskrebs/gebaermutterhalskrebs.html. Accessed 09 June 2022

[15] Cuzick J, Szarewski A, Cubie H (2003). Management of women who test positive for high-risk types of human papillomavirus: the HART study. Lancet.

[16] Sherman ME, Lorincz AT, Scott DR (2003). Baseline cytology, human papillomavirus testing, and risk for cervical neoplasia: a 10-year cohort analysis. J Natl Cancer Inst.

[17] Bulkmans NW, Berkhof J, Rozendaal L (2007). Human papillomavirus DNA testing for the detection of cervical intraepithelial neoplasia grade 3 and cancer: 5-year follow-up of a randomised controlled implementation trial. Lancet.

[18] Mayrand MH, Duarte-Franco E, Rodrigues I (2007). Human papillomavirus DNA versus Papanicolaou screening tests for cervical cancer. N Engl J Med.

[19] Ronco G, Giorgi-Rossi P, Carozzi F (2010). Efficacy of human papillomavirus testing for the detection of invasive cervical cancers and cervical intraepithelial neoplasia: a randomised controlled trial. Lancet Oncol.

[20] Elfstrom KM, Smelov V, Johansson AL (2014). Long term duration of protective effect for HPV negative women: follow-up of primary HPV screening randomised controlled trial. BMJ.

[21] Horn J, Denecke A, Luyten A (2019). Reduction of cervical cancer incidence within a primary HPV screening pilot project (WOLPHSCREEN) in Wolfsburg. Germany Br J Cancer.

[22] https://www.g-ba.de/themen/methodenbewertung/ambulant/frueherkennung-krankheiten/erwachsene/krebsfrueherkennung/gebaermutterhalskrebs-screening/. Accessed 9 June 2022

[23] Katki HA, Kinney WK, Fetterman B (2011). Cervical cancer risk for women undergoing concurrent testing for human papillomavirus and cervical cytology: a population-based study in routine clinical practice. Lancet Oncol.

[24] Arbyn M, Simon M, Peeters E (2021). 2020 list of human papillomavirus assays suitable for primary cervical cancer screening. Clin Microbiol Infect.

[25] Meijer CJ, Berkhof J, Castle PE (2009). Guidelines for human papillomavirus DNA test requirements for primary cervical cancer screening in women 30 years and older. Int J Cancer.

[26] Bornstein J, Bentley J, Bosze P (2012). 2011 colposcopic terminology of the International federation for cervical pathology and colposcopy. Obstet Gynecol.

[27] [Anonym]. Leitlinienprogramm Onkologie (Deutsche Krebsgesellschaft, Deutsche Krebshilfe,AWMF): Prävention des Zervixkarzinoms, Langversion 1.1, 2020, AWMF Registernummer: 015/027OL, http://www.leitlinienprogramm-onkologie.de/leitlinien/zervixkarzinom-praevention/ (abgerufen am: 19.03.2022). DOI:

[28] Akaike H (1974). A new look at the statistical model identification. IEEE Trans Autom Control.

[29] Hothorn THK, Zeileis A (2006). Unbiased recursive partitioning: a conditional inference framework. J Comput Gr Stat.

[30] R Core Team (2019) R: A language and environment for statistical computing. R Foundation for Statistical Computing V, Austria.https://www.R-project.org/. Accessed 9 June 2022

[31] Liu Y, Liao J, Yi X (2022). Diagnostic value of colposcopy in patients with cytology-negative and HR-HPV-positive cervical lesions. Arch Gynecol Obstet.

[32] Underwood M, Arbyn M, Parry-Smith W (2012). Accuracy of colposcopy-directed punch biopsies: a systematic review and meta-analysis. BJOG.

[33] Zuchna C, Hager M, Tringler B (2010). Diagnostic accuracy of guided cervical biopsies: a prospective multicenter study comparing the histopathology of simultaneous biopsy and cone specimen. Am J Obstet Gynecol.

[34] Petousis S, Christidis P, Margioula-Siarkou C (2018). Discrepancy between colposcopy, punch biopsy and final histology of cone specimen: a prospective study. Arch Gynecol Obstet.

[35] Cantor SB, Cardenas-Turanzas M, Cox DD (2008). Accuracy of colposcopy in the diagnostic setting compared with the screening setting. Obstet Gynecol.

[36] Davies KR, Cantor SB, Cox DD (2015). An alternative approach for estimating the accuracy of colposcopy in detecting cervical precancer. PLoS ONE.

[37] Barut MU, Kale A, Kuyumcuoglu U (2015). Analysis of sensitivity, specificity, and positive and negative predictive values of smear and colposcopy in diagnosis of premalignant and malignant cervical lesions. Med Sci Monit.

[38] Fatahi Meybodi N, Karimi-Zarchi M, Allahqoli L (2020). Accuracy of the triple test versus colposcopy for the diagnosis of premalignant and malignant cervical lesions. Asian Pac J Cancer Prev.

[39] Mousavi AS, Fakour F, Gilani MM (2007). A prospective study to evaluate the correlation between Reid colposcopic index impression and biopsy histology. J Low Genit Tract Dis.

[40] Sesti F, Farne C, Mattei M (1990). Role of endocervical curettage in the diagnostic workup of preinvasive cervical lesions. Int J Gynaecol Obstet.

[41] Li Y, Luo H, Zhang X (2021). Development and validation of a clinical prediction model for endocervical curettage decision-making in cervical lesions. BMC Cancer.

[42] Hashim D, Engesaeter B, Skare GB (2020). Real-world data on cervical cancer risk stratification by cytology and HPV genotype to inform the management of HPV-positive women in routine cervical screening. Brit J Cancer.

[43] Katki HA, Schiffman M, Castle PE (2013). Benchmarking CIN 3+risk as the basis for incorporating HPV and Pap cotesting into cervical screening and management guidelines. J Low Genit Tract Di.

[44] Richardson LA, El-Zein M, Ramanakumar AV (2015). HPV DNA testing with cytology triage in cervical cancer screening: influence of revealing HPV infection status. Cancer Cytopathol.

[45] Bergeron C, Giorgi-Rossi P, Cas F (2015). Informed cytology for triaging HPV-positive women: substudy nested in the NTCC randomized controlled trial. J Natl Cancer Inst.

[46] Wentzensen N, Schiffman M, Palmer T (2016). Triage of HPV positive women in cervical cancer screening. J Clin Virol.

[47] Khan MJ, Castle PE, Lorincz AT (2005). The elevated 10-year risk of cervical precancer and cancer in women with human papillomavirus (HPV) type 16 or 18 and the possible utility of type-specific HPV testing in clinical practice. J Natl Cancer Inst.

[48] Kjaer SK, Frederiksen K, Munk C (2010). Long-term absolute risk of cervical intraepithelial neoplasia grade 3 or worse following human papillomavirus infection: role of persistence. J Natl Cancer Inst.

[49] Munmany M, Marimon L, Cardona M (2017). Small lesion size measured by colposcopy may predict absence of cervical intraepithelial neoplasia in a large loop excision of the transformation zone specimen. BJOG.

